# *Drosophila* TRPN( = NOMPC) Channel Localizes to the Distal End of Mechanosensory Cilia

**DOI:** 10.1371/journal.pone.0011012

**Published:** 2010-06-08

**Authors:** Jeongmi Lee, Sungjin Moon, Yoonseok Cha, Yun Doo Chung

**Affiliations:** Department of Life Sciences, University of Seoul, Seoul, Korea; Institute for Research in Biomedicine, Spain

## Abstract

**Background:**

A TRPN channel protein is essential for sensory transduction in insect mechanosensory neurons and in vertebrate hair cells. The *Drosophila* TRPN homolog, NOMPC, is required to generate mechanoreceptor potentials and currents in tactile bristles. NOMPC is also required, together with a TRPV channel, for transduction by chordotonal neurons of the fly's antennal ear, but the TRPN or TRPV channels have distinct roles in transduction and in regulating active antennal mechanics. The evidence suggests that NOMPC is a primary mechanotransducer channel, but its subcellular location—key for understanding its exact role in transduction—has not yet been established.

**Methodology/Principal Findings:**

Here, by immunostaining, we locate NOMPC at the tips of mechanosensory cilia in both external and chordotonal sensory neurons, as predicted for a mechanotransducer channel. In chordotonal neurons, the TRPN and TRPV channels are respectively segregated into distal and proximal ciliary zones. This zonal separation is demarcated by and requires the ciliary dilation, an intraciliary assembly of intraflagellar transport (IFT) proteins.

**Conclusions:**

Our results provide a strong evidence for NOMPC as a primary transduction channel in *Drosophila* mechansensory organs. The data also reveals a structural basis for the model of auditory chordotonal transduction in which the TRPN and TRPV channels play sequential roles in generating and amplifying the receptor potential, but have opposing roles in regulating active ciliary motility.

## Introduction

Mechanically-activated ion channels are presumed to generate fast receptor potentials in the sensory cells that transduce touch and sound, but establishing the molecular identity of these channels has been problematic. Even when strong transducer channel candidates have been identified, as in nematode touch cells and insect bristles, the mechanism by which these channels open is still unclear. However, mechanoreceptor organs are typically highly structured, with extracellular and cytoskeletal structures adapted to transmit mechanical stimuli to the sensory endings where transduction occurs. Determining the location of candidate transducer channels within or relative to these structures can help to confirm the function of the channels and to understand how they are activated.

The TRPN proteins are strong candidates for mechanotransducer channel subunits in both vertebrates and invertebrates. TRPN homologs are present in insects, nematodes, fish and amphibians [1], [2], [3], [4], and are required for tactile and proprioceptive behavior in insects and nematodes [1], [5], and for transduction of vibratory stimuli by zebrafish hair cells [4]. By sequence analysis, they form a distinct subgroup within the TRP channel superfamily, and are also distinguished by a highly conserved, N-terminal, cytoplasmic array of 28–29 ankyrin repeats.

A TRPN protein was first identified in *Drosophila* as the site of *no mechanoreceptor potential C* (*nompC*) mutations [1], which affect ciliated mechanoreceptors. These include external sensory organs such as bristles and campaniform sensilla, and internal chordotonal organs. An individual sensillum or a chordotonal sensory unit comprises several specialized support cells and one to three sensory neurons. Each neuron has a single sensory process or inner segment, tipped by a ciliary outer segment where the initial transduction event is thought to occur [6]. *nompC* null mutants lack adapting mechanoreceptor potentials and currents in tactile bristles [1], [7], and a missense allele, *nompC^4^*, increases the adaptation kinetics of the receptor current [1], suggesting that the NOMPC protein is an integral part of the transducer machinery.

*nompC* mutants also show severely reduced sound-evoked potentials in Johnston's organ (JO), a large antennal chordotonal organ which transduces vibrations from near-field sound sources [8]. Antennal sound-evoked potentials also require Nanchung (NAN) and Inactive (IAV), subunits of a TRPV channel located in the chordotonal cilia [9], [10]. A non-linear compliance and active oscillation of the antennae, which appear to originate in JO, amplify its response to weak stimuli [11], [12]. The oscillations are reduced in *nompC* mutants but are greatly increased in *iav* and *nan* mutants, indicating that the NOMPC and TRPV channels normally have opposing roles in regulating it [13].

Thus the genetic and physiological evidence suggests that NOMPC does form a primary mechanotransducer channel, but without a molecular marker for the protein, its exact location and role in transduction have been uncertain. Here we report that an antiserum to a NOMPC fragment labels ciliary foci or zones in both bristles and chordotonal organs, in wild type but not in *nompC* null mutants. In chordotonal organs, NOMPC and IAV proteins show a striking segregation into distal and proximal ciliary zones respectively, revealing a structural basis for the distinct roles of the TRPN and TRPV channels in sensory transduction and amplification.

## Results

### NOMPC is located at the distal tips of sensory cilia in tactile bristles

**Figure 1:**
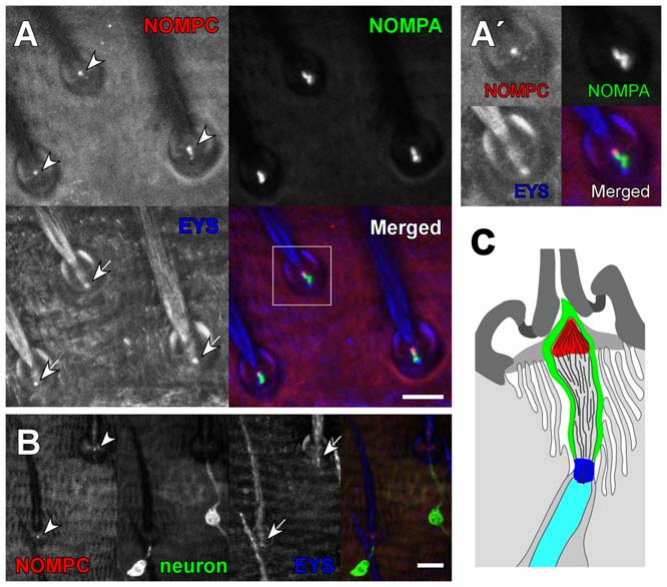
NOMPC localizes in the distal tip of sensory cilia in tactile bristles. **A–B:** Mechanosensory bristles on the dorsal abdomen, immunostained with anti-NOMPC antiserum (red). The monoclonal antibody 21A6, which detects the extracellular protein EYS, marks the proximal end of the sensory cilia (blue, arrows). Dendritic sheaths were labeled with GFP::NOMPA (green in **A**), and the sensory neurons were visualized by expressing membrane-associated CD8::GFP (green in **B**). The dot-shaped NOMPC signals (arrowheads) are seen only at the distal tips of the sensory cilia, and are clearly separated from the EYS signals (arrows). The enlarged images of the area marked by a rectangle in **A** are shown in **A'**. **C:** Schematic drawing of an abdominal tactile bristle. Green represents the dendritic cap, cyan the inner dendritic segment. Red and blue represent the area labeled by anti-NOMPC and mAb 21A6 respectively. Scale bars represent 10 µm.

**Figure 2:**
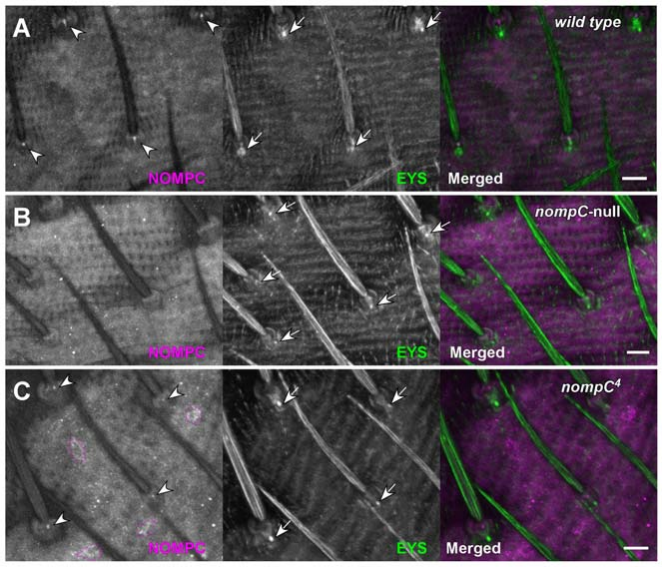
NOMPC localization in abdominal bristles from wild type or *nompC* mutants. Abdominal cuticles from wild type (**A**) or *nompC* mutants (**B–C**) pharate adults were doubly stained with anti-NOMPC (magenta) and anti-EYS (mAb 21A6, green) antibodies. Focal anti-NOMPC signals at bristle bases (arrowheads in **A**) were seen in wild type, but not in *nompC* null (**B**). In *nompC^4^* (**C**), reduced NOMPC foci are present in the bristle bases (arrow heads in **C**), distal to the EYS foci, as in wild type; but a significant amount of signal is also seen in the cell body region (outlined by dotted lines in **C**). Scattered non-specific signals are seen in both wild type and mutant cuticles. The arrows indicate anti-EYS staining at the basal end of the sensory cilia. Note: the gain of magenta channel in **B** and **C** is much higher than that in **A**. Scale bars represent 10 µm.

A cytoplasmic, N-terminal segment (amino acids 14–117) preceding the ankyrin repeat domain of NOMPC was expressed as a GST fusion protein and used to generate two rabbit antisera. Several alternately spliced isoforms of the transcript are predicted and observed in cDNA clones [ref. 1; Flybase (http://www.flybase.org)], but all isoforms share this N-terminal coding sequence and should be detected by these antisera. When used to immunostain wild type pupal cuticle, both antisera labelled a dot at the base of each mechanosensory bristle. Figure 1 shows the confocal images obtained using one of the antisera. No similar signal was detected in *nompC*-null mutants (Figure 2B) indicating that this focal signal represents a *nompC* gene product. In homozygous for *nompC^4^*, a missense mutation which retains the bristle receptor current but changes its adaptation kinetics, the focal signal is present but reduced in intensity, and some punctuate labeling is seen in the neuronal cell body (Figure 2C).

To determine the precise subcellular location of NOMPC, we examined preparations labeled for two previously described extracellular sensory proteins, the agrin/perlecan-related protein Eyes Shut (EYS) or Spacemaker (SPAM) [14], [15], [16] and the zona pellucida (ZP) domain protein NOMPA [17]. The ciliary outer segments of bristle sensory processes are divided into two structurally distinct sections: a proximal connecting cilium with a short axoneme, and a longer, distal section filled with an array of densely packed microtubules, the tubular body; EYS/SPAM fills the extracellular space surrounding the connecting cilium [17]. Double-labeling with anti-NOMPC and mAb 21A6, which detects EYS, showed the NOMPC focus to be separate from and distal to the EYS signal, placing it in the tubular body section, not in the connecting cilium (Figure 1).

The distal part of the cilium is enclosed by a thin sheath of electron-dense extracellular matrix, which includes NOMPA [6], [17]. Flies expressing a functional, GFP-tagged NOMPA protein from a native transgene construct show an extended patch of GFP labeling at each bristle, outlining the sheath and the cilium. The anti-NOMPC signal was concentrated at the end of this NOMPA-GFP label closer to the bristle base, indicating that NOMPC is localized at the apical tip of the cilium (see the schematic drawing in Figure 1C).

### Segregation of NOMPC and TRPV channels in chordotonal cilia

Chordotonal organs are internal stretch and vibration receptors composed of sensory units called scolopidia. Each scolopidium includes one to three sensory neurons whose ciliary outer segments are enclosed in a fluid-filled capsule, the scolopale. Chordotonal cilia have an extended axonemal structure, with a cylinder of nine microtubule doublets extending almost their full length. Midway along each cilium is a ciliary dilation (CD), where the membrane and the axonemal microtubules bulge outward to enclose an electron-dense inclusion. The tips of the cilia in each scolopidium are attached to an extracellular cap, which like the sheath of a bristle cilium includes NOMPA. The cap, however, contacts only the distal third of the cilium. The EYS protein accumulates in the scolopale space in aggregations around the middle and the base of the cilium [14], [16].

**Figure 3:**
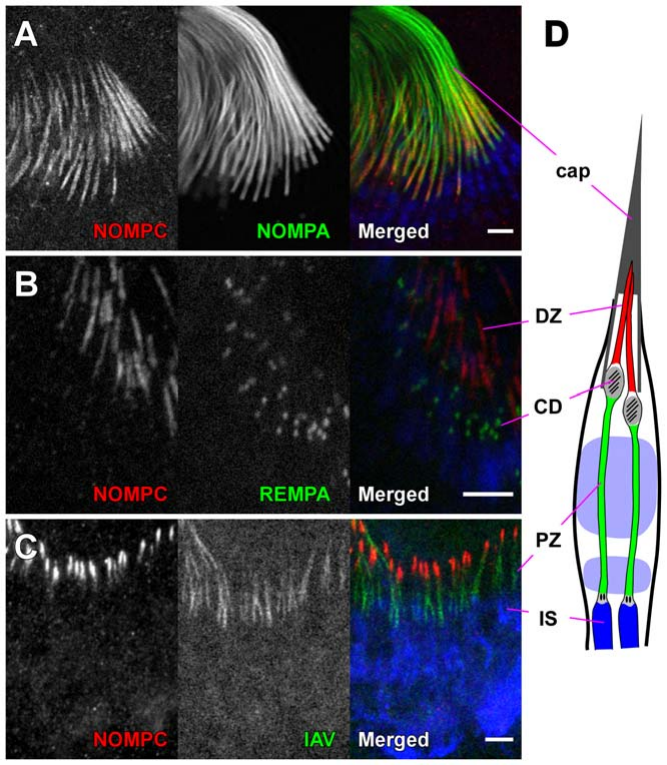
Localization of NOMPC and segregation from TRPV channels in chordotonal cilia. Whole-mount staining of antennal chordotonal organs from late pupae, labeled with anti-NOMPC and counterstained either with mAb 21A6 (**A**–**B**), which labels the scolopale space, or with 22C10 (**C**), which labels the neuronal cell bodies and inner dendritic segments, but not cilia. The different labelled structures are shown in a schematic (**D**). NOMPC labeling coincides with the dendritic caps, which are labeled with expressed GFP-NOMPA (green in **A**). NOMPC is restricted distal to the ciliary dilation, labeled with IFT140/REMPA-YFP (green in **B**). A ciliary TRPV channel, labeled with expressed IAV-GFP (green in **C**), is restricted to a zone proximal to the NOMPC label. cap: dendritic cap; CD: ciliary dilation; DZ: distal ciliary zone; PZ: proximal ciliary zone. Scale bars represent 5 µm.

**Figure 4:**
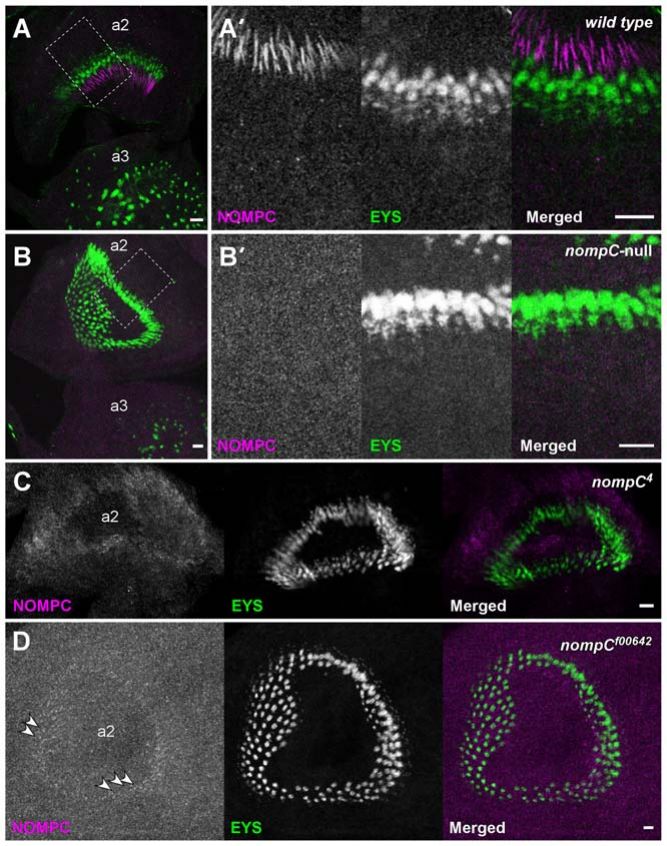
Altered localization or expression of NOMPC in JO from *nompC* mutants. Pupal antennae in wild type (**A**) or *nompC* mutants (**B–D**) were stained with anti-NOMPC (magenta) and anti-EYS (green) antibodies. In wild type (**A** and **A'**), NOMPC is detected in the chordotonal cilia of JO in the 2nd antennal segment (a2). No NOMPC signals are seen in any antennal region of the *nompC* null mutant (**B** and **B'**). Dotted rectangles in **A** and **B** indicate the enlarged areas shown in **A'** and **B'**, respectively. In *nompC^4^* mutant (**C**), the ciliary NOMPC pattern is eliminated; NOMPC signals are detected only in the region of the cell bodies. In *nompC^f00642^* mutant (**D**), NOMPC is still localized at the distal cilia (arrows in **D**), but greatly reduced. Note: the gain of magenta channel in **D** is much higher than in other panels. EYS surrounds the proximal zone of the cilia. a2: 2nd antennal segment; a3: 3rd antennal segment. Scale bars represent 5 µm.

We examined NOMPC localization in JO, the antennal auditory organ which comprises hundreds of scolopidia. NOMPC immunolabeling was concentrated in the distal part of the cilia, specifically in the zone enclosed by the NOMPA-labelled cap (Figure 3A). No similar signals were detected in *nompC*-null mutants, confirming again the specificity of the antibody (Figure 4B). Interestingly, *nompC^4^* mutants also showed no detectable anti-NOMPC signal in chordotonal cilia, although immunolabeling was visible in the region of the neuronal cell bodies (Figure 4C). This contrasts with the retention of the mutant protein in bristle cilia, but is consistent with the effects of *nompC* null and *nompC^4^* mutations on chordotonal transduction: both greatly reduce but do not eliminate sound-evoked antennal potentials (data not shown). We also examined the antennae of a new hypomorphic mutant, *nompC^f00642^*, in which the amount of correctly spliced *nompC* mRNA is reduced by over 90% [18]. In this mutant, as consistent with the mRNA level, NOMPC immunoreactivity was greatly reduced, although it was still concentrated in the distal zone of sensory cilia (Figure 4D), confirming further the specificity of our antibody.

We examined the location of NOMPC relative to REMPA, the *Drosophila* homolog of the intraflagellar transport protein IFT140, which is localized at the CD and is required for the normal organization of the cilium and the CD [19]. In flies expressing a functional REMPA-YFP fusion [19], the CD is the proximal limit of NOMPC labeling (Figure 3B). The TRPV channel that includes the Nanchung and Inactive subunits was previously shown to be localized proximal to the CD [9], suggesting that it and NOMPC have a complementary distribution. Indeed, double-labeling of antennae with anti-NOMPC and a functional GFP-tagged IAV, clearly shows that the two channel proteins are segregated into distinct ciliary regions: NOMPC in the distal part and IAV in the proximal part (Figure 3C **)**. A similar segregation is also shown by embryonic chordotonal neurons (Figure S1).

**Figure 5:**
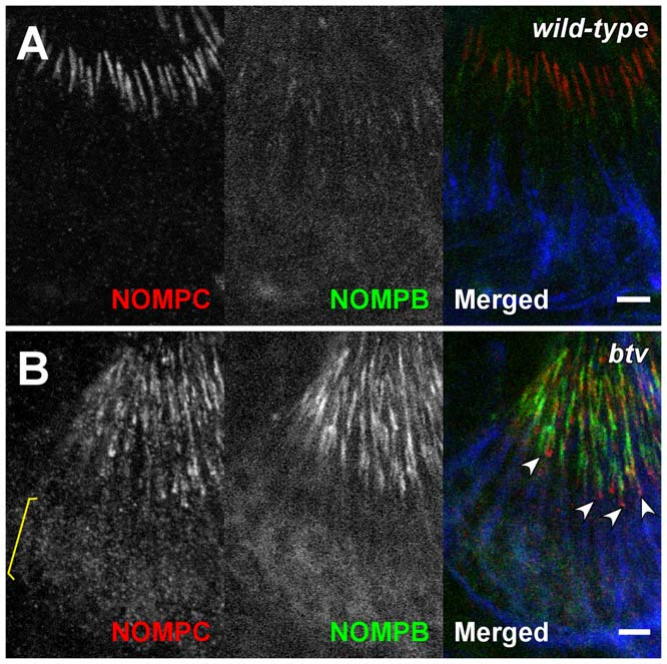
NOMPC is mislocalized in *btv* mutant cilia. Antennal chordotonal neurons of wild type (**A**) or *btv* mutant (**B**) were stained with anti-NOMPC antiserum (red). Cilia were labeled with a functional GFP-tagged NOMPB/IFT88 [24] (green). The neuronal cell bodies and inner segments were counter-stained with mAb 22C10 (blue). NOMPB/IFT88 is normally detected only in the proximal segment, whereas NOMPC is only in the distal segment of the sensory cilia (**A**). In *btv*, however, NOMPB/IFT88 and NOMPC distributions overlap along the whole cilia, especially accumulating at the base of cilia (arrow heads) (**B**). NOMPC signal is also seen in cell body regions (yellow bracket in **B**). Note: the ciliary accumulation of NOMPB/IFT88 is much higher in *btv* mutant than wild type. Scale bars represent 5 µm.

In *beethoven* (*btv*) mutant flies, which lack the IFT-associated dynein heavy chain, the CD is disrupted [8], and the TRPV channel is mislocalized, with some of the TRPV channel found in the distal zone [19]. The distribution of NOMPC is also affected: NOMPC is delocalized and present in the proximal zone from which it is normally excluded (Figure 5). Thus, IFT dynein activity and/or an intact ciliary dilation are required to maintain the segregation of the TRPN and TRPV channels.

### NOMPC is expressed by almost all chordotonal sensory units in JO

**Figure 6:**
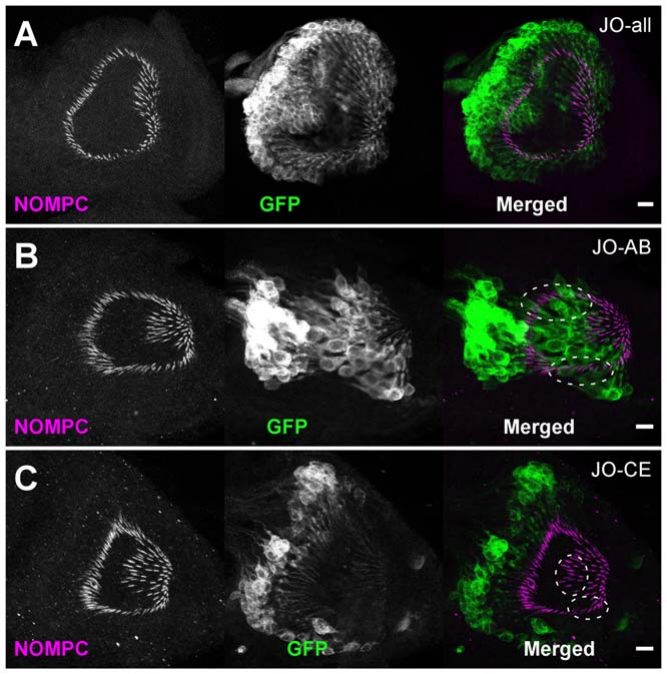
NOMPC is expressed by almost all Chordotonal organs of JO. Confocal projection images of antennal chordotonal organs from late pupae expressing CD8::GFP driven by JO subgroup-specific GAL4 drivers. In JO-all flies in which almost all antennal chordotonal neurons express GAL4-driven GFP, almost every GFP-expressing neuron has NOMPC signal (magenta) at its distal cilium (**A**). In flies expressing GAL4-driven GFP only in JO subgroups AB (**B**) or CE (**C**) neurons, however, NOMPC signals are not only restricted in GFP-expressing cilia but also detected outside of GFP-expressing neurons (dotted ovals in **B** and **C**). Genotypes: *UAS-CD8::GFP/+; f-GAL4/+* (**A**), *UAS-CD8::GFP/+; JO15/+* (**B**), *UAS-CD8::GFP/+; NP6250/+* (**C**). Scale bars represent 10 µm.

It has recently been reported that JO comprises several different subsets of chordotonal neurons which project their axons to distinct zones of the brain's antennal mechanosensory and motor centre (AMMC) [20], [21]. Calcium imaging showed that different sensory stimuli are transduced by different subsets of JO neurons, and each sensory information travels in parallel to separate zones in the AMMC. The subgroups AB neurons, which are rapidly adapting and vibration sensitive, are activated by sound. In contrast, the slowly adapting, deflection-sensitive neurons (subgroups CE neurons) are responsible for gravity and wind sensing [20], [21]. Analysis of GAL4 expression under the control of *nompC* promoter suggested that *nompC* seems to be expressed only by the sound-sensitive neurons, indicating that gravity (or wind) sensing is independent of NOMPC [20]. In consistent with this finding, *nompC^f00642^* mutant showed normal behavioral response to gravity, whereas the sound-evoked antennal potentials are reduced [18]. To our surprise, however, NOMPC immunoreactivities were not restricted in the subgroups AB neurons, but detected in almost all chordotonal sensory units (Figure 6), indicating that the *nompC-GAL4* driver does not represent the whole repertoire of *nompC*-expressing cells. This also suggests that NOMPC may have a role in both sound- and gravity-sensitive neurons.

## Discussion

The focal and zonal signals labeled by our antiserum represent endogenous NOMPC gene products, as evidenced by their absence from *nompC* null mutants and reduction in *nompC^f00642^* mutant, and by the mislocalized label in the *nompC^4^* missense mutant. They may represent any or all of the predicted alternate spliceforms, all of which include the peptide sequence used as an antigen. The data support a role for NOMPC as a primary transducer channel, because it is located at sites where mechanical signals impinge on the sensory cilia in both external and chordotonal organs. In both receptor types, this is at a site where the ciliary membrane is contacted by the extracellular matrix of the dendritic cap or sheath. This could mean that matrix elements are direct ligands for the TRPN channel. Indeed, *nompA* mutations detach the cilium from the matrix and eliminate transduction [17]. This is not seen in *nompC* null mutants [1], so the channel cannot be the only connection between the cilium and cap. This is also consistent with the limited extracellular exposure of the NOMPC channel's predicted topology. However, no direct interaction has been demonstrated between any identified mechanosensory channel and an extracellular protein or matrix; it is also possible that the sensory transducers are basically stretch-activated channels and that the specialized extracellular and cytoskeletal structures are required to activate the channel indirectly by locally increasing membrane tension.

From a detailed analysis of antennal mechanics, sequential roles for TRPN and TRPV in fly auditory transduction have been proposed [13]. In this model, TRPN is the primary transduction channel that triggers the mechanical amplification of the antennal vibrations, while TRPV functions as a secondary channel required for the generation of action potentials but also downregulates the antennal vibrations [for review see ref. 6]. A puzzle is how two TRP channels, both probably cation-selective, have distinct effects in the same cell. Their spatial separation provides one possible explanation: the CD may divide the sensory cilium into two functionally distinct zones in chordotonal neurons. In support of this idea, the two zones also differ in their axoneme structures: axonemal dynein-like arms are found only in the proximal zone [19], [22]. It seems likely that the non-motile distal segment is the place where the initial mechanotransduction current occurs, whereas the potentially motile proximal segment amplifies the sound stimuli by actively vibrating the cilium in response to the initial transduction current. TRPV in proximal segment may act as a secondary channel that generates depolarizing current needed to trigger the action potentials. Opening of TRPV may also negatively regulate the active vibration of the proximal segment.

In this study, the antibody staining showed that NOMPC is expressed in almost all sensory units of JO, although it is unclear whether every neuron in each sensory unit expresses NOMPC. This result contrasts with the previous report that argued the subgroup-specific expression of NOMPC on the basis of the expression pattern of GAL4 under the control of *nompC* promoter [20]. But the promoter-fusion construct does not necessarily represent the whole expression pattern of the endogenous gene. Indeed, the *nompC-GAL4* does not express GAL4 in sensory neurons of abdominal bristles (Figure S2) in which NOMPC is required for mechanosensory transduction [1], [7]. Thus it is reasonable to conclude that NOMPC is expressed in all sensory units of JO, and participated in sensing not only sound but also gravity and wind. Further functional analyses are required to confirm this.

In some vertebrates, TRPN channels also have an essential role in sensory hair cell function [3], [4]. Most hair cells include both a true cilium, the kinocilium, and a bundle of actin-based sterecilia that are the probable site of mechanosensory transduction. In *Xenopus*, TRPN1 localizes only to kinocilium, particularly to its bulbous tip [3]. This suggests that the essential role for frog TRPN1 is in the kinocilium instead of main transduction channel. However, the precise role of TRPN1 in the frog kinocilium is unclear. In zebrafish, TRPN1 is also required for hair cell function [4]; but its precise role and subcellular localization in hair cells is not determined yet. It is of interest to note that some fish kinocilia can beat spontaneously or in response to tip stimulation [23]. It will be interesting to examine whether the ciliary motility in vertebrate hair cell transduction requires TRPN1.

## Materials and Methods

### Flies

Transgenic lines expressing NOMPA::GFP and IAV::GFP were described previously [9], [17]. Flies expressing REMPA::YFP and *btv* mutant flies expressing NOMPB::GFP (*btv^5P1^*/*Cy GFP; GFP-NOMPB*) were obtained from M. Kernan (Stony Brook University, NY, USA). *nompC*-null flies were generated by selecting *nompC^1^*/*nompC^2^* transheterozygotes from the cross: *nompC^1^*/*Cy* × *nompC^2^*/*Cy*. M. Kernan also provided the *nompC* mutant lines including *nompC^1^*, *nompC^2^* and *nompC^4^*. The missense point mutation in *nompC^4^* was confirmed by sequencing. The *nompC^f00642^* flies were provided by M. Welsh (University of Iowa, IA, USA). The subset-specific JO GAL4 lines described in the previous paper [20] were obtained from Bloomington (Bloomington, IN, USA) or DGRC (Kyoto, Japan) stock centers.

### Antibodies

A cDNA fragment encoding the N-terminal region of *Drosophila* NOMPC (amino acids 14–117) was amplified by PCR and cloned into pGEX4T3 vector to produce GST-fusion protein. Cloning, expression and purification of the GST-fusion protein were according to the GST Gene Fusion System Handbook (GE healthcare, Buckinghamshire, UK). The purified fusion protein was injected into two rabbits to produce anti-NOMPC antisera. One of the antisera, after affinity purification, was used in this study. For immunostaining, tissue samples were stained with the purified anti-NOMPC antiserum at 1∶50 ∼ 1∶100 dilution. The monoclonal antibodies used in this study (21A6 and 22C10) were purchased from the Developmental Studies Hybridoma Bank (DSHB, http://dshb.biology.uiowa.edu/), and used at 1∶50 ∼ 1∶100 dilution.

### Immunostaining and confocal microscopy

Embryos, pupal antennae and pharate adult abdominal cuticles were prepared for whole mount staining as described previously [17]. This included fixation in 4% formaldehyde in PBS (10 mM NaPO_4_ (pH 7.2), 150 mM NaCl) for 15 min, three 10-minutes washes in PBT (PBS + 0.1% Triton X-100), blocking for 1 hr in 2% normal goat serum + 2% BSA in PBT, and incubation with primary antibodies overnight at 4°C in the blocking solution. Then, after three 10-minute washes in PBT, samples were incubated with secondary antibodies for 2 hrs at room temperature, washed three times in PBT for 10 minutes each, and mounted in 80% glycerol. Samples were imaged with a laser scanning confocal microscope (Carl Zeiss, LSM510). The following secondary antibodies were obtained from Invitrogen (Carlsbad, CA, USA) and used at the dilutions indicated: Alexa-633 conjugated goat anti-mouse (1∶2,000), Alexa-543 conjugated goat anti-rabbit (1∶500).

## Supporting Information

Figure S1 **NOMPC localization in developing chordotonal organs. A:** A pentascolopidial chordotonal organ in a stage 14 embryo. NOMPC immunoreactivity (magenta) is detected both in cell bodies and in sensory cilia at this stage. **B:** At late embryonic stage (stage 17), NOMPC signals are enriched in the distal zones of sensory cilia, but only faint signals are seen in cell body. Sensory neurons were also labeled with mAb 22C10 (green), which stains neuronal cell bodies and inner dendritic segments, but not cilia. **C:** Interpretive schematic drawing of a larval chordotonal organ, showing NOMPC (magenta) in the distal zone of the sensory cilium, but not in the proximal zone. cap: dendritic cap; CD: ciliary dilation; DZ: distal ciliary zone; PZ: proximal ciliary zone; SC: sensory cilium. Scale bars represent 10 μm.
(0.30 MB JPG)

Figure S2 **The *nompC-GAL4*-driven GFP is not expressed in the sensory neurons of tactile bristles. A:** Adult abdominal bristles expressing CD8::GFP under the control of elav-GAL4, which expresses GAL4 in every neuron. The GFP signals (Green) are detected in all the sensory neurons (arrow heads) that innervate the bristles (arrows). In each bristle, only a single sensory neuron is associated with the base. **B:** Adult abdominal bristles expressing CD8::GFP under the control of *nompC-GAL4*. No GFP-expressing neurons are seen in the bristles (arrows). The GFP signals are detected only in some non-neuronal cells (asterisks). Scale bars represent 10 μm.
(0.25 MB JPG)
