# Supplementary figures and images for: Drosophila TRPN( = NOMPC) Channel Localizes to the Distal End of Mechanosensory Cilia
Source: PLoS One. 2010 Jun 8;5(6):e11012. doi: 10.1371/journal.pone.0011012 (PMC2882365; doi:10.1371/journal.pone.0011012)

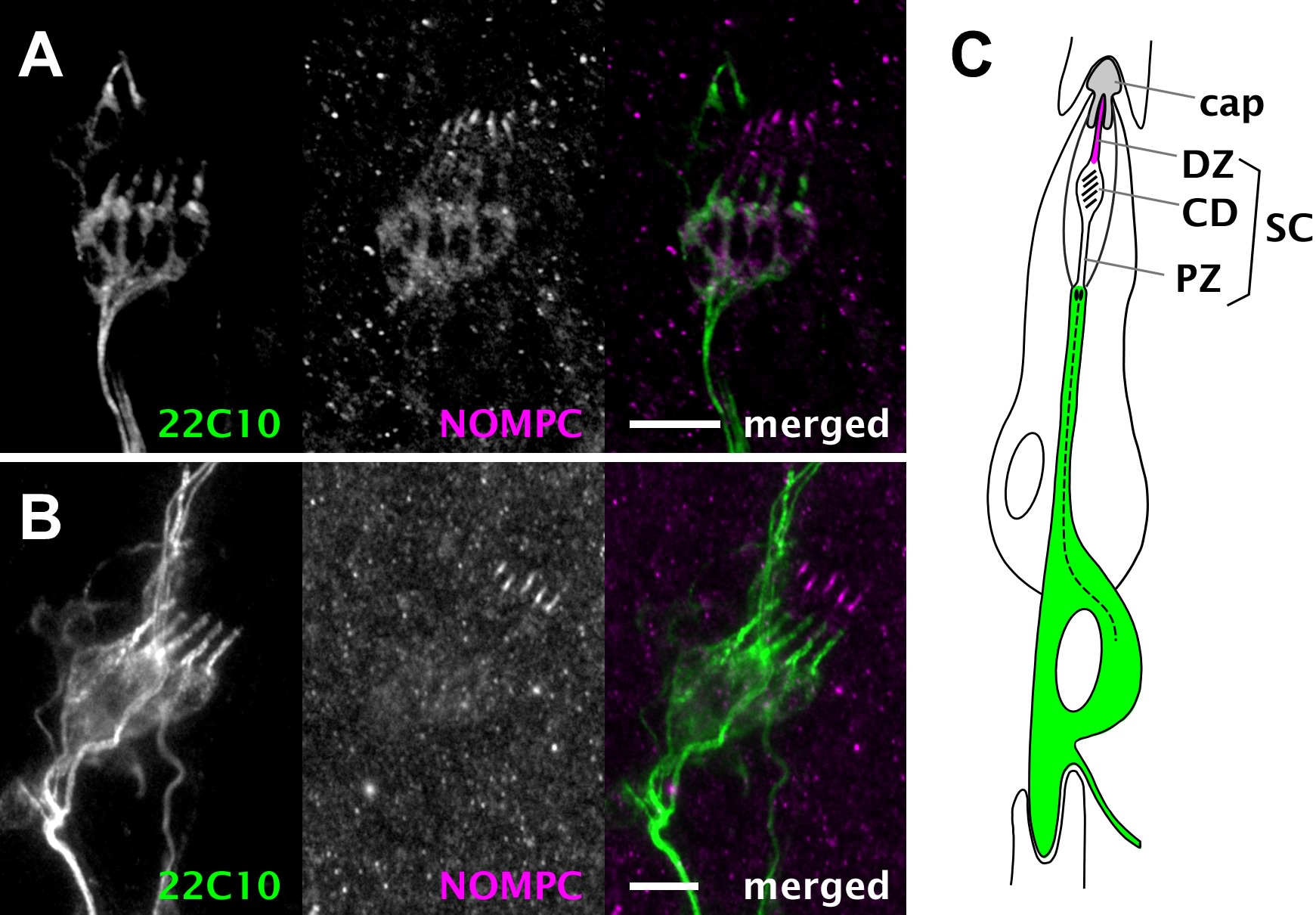

Supplement: Figure S1 — NOMPC localization in developing chordotonal organs. A: A pentascolopidial chordotonal organ in a stage 14 embryo. NOMPC immunoreactivity (magenta) is detected both in cell bodies and in sensory cilia at this stage. B: At late embryonic stage (stage 17), NOMPC signals are enriched in the distal zones of sensory cilia, but only faint signals are seen in cell body. Sensory neurons were also labeled with mAb 22C10 (green), which stains neuronal cell bodies and inner dendritic segments, but not cilia. C: Interpretive schematic drawing of a larval chordotonal organ, showing NOMPC (magenta) in the distal zone of the sensory cilium, but not in the proximal zone. cap: dendritic cap; CD: ciliary dilation; DZ: distal ciliary zone; PZ: proximal ciliary zone; SC: sensory cilium. Scale bars represent 10 μm. (0.30 MB JPG) [file pone.0011012.s001.jpg]

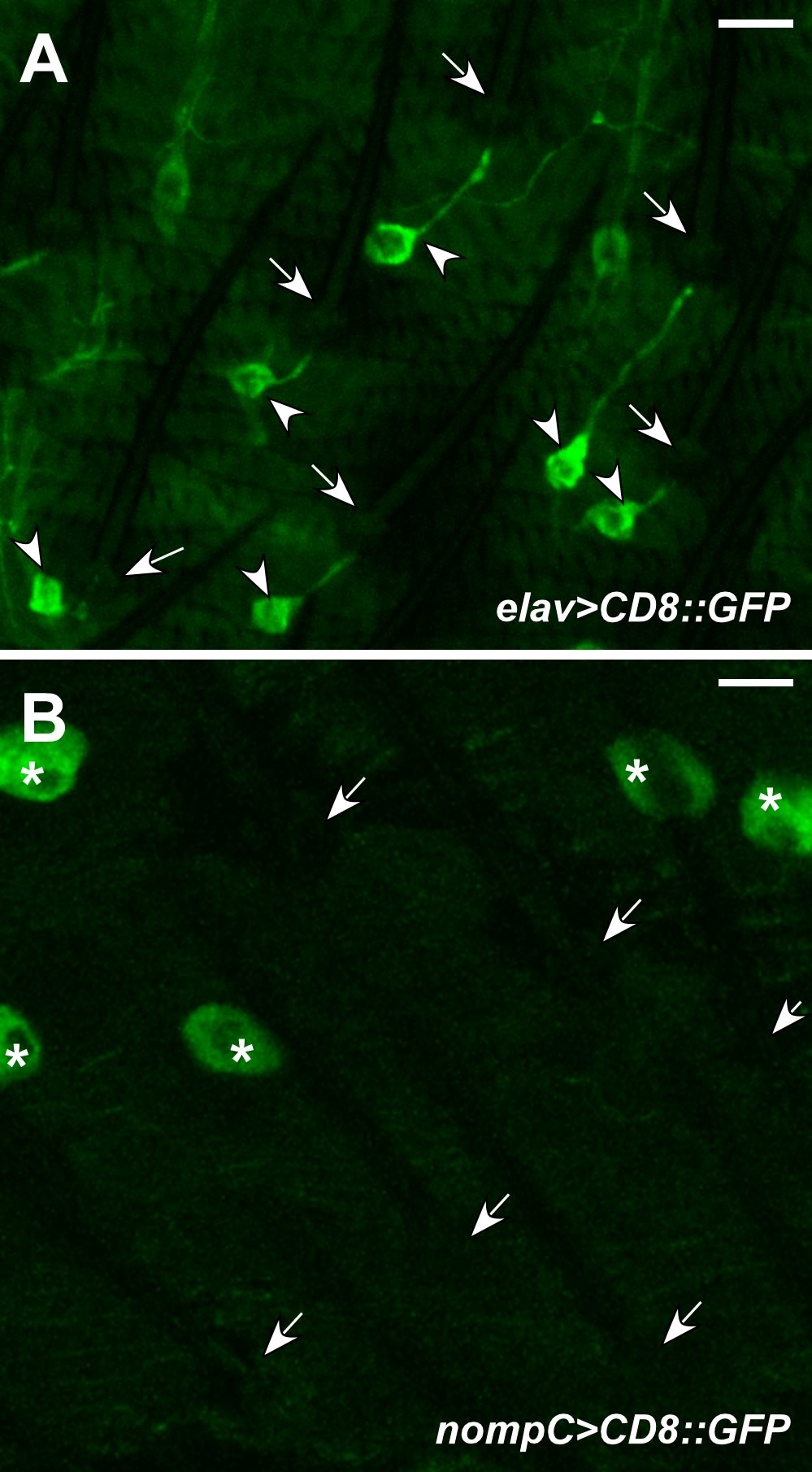

Supplement: Figure S2 — The nompC-GAL4-driven GFP is not expressed in the sensory neurons of tactile bristles. A: Adult abdominal bristles expressing CD8::GFP under the control of elav-GAL4, which expresses GAL4 in every neuron. The GFP signals (Green) are detected in all the sensory neurons (arrow heads) that innervate the bristles (arrows). In each bristle, only a single sensory neuron is associated with the base. B: Adult abdominal bristles expressing CD8::GFP under the control of nompC-GAL4. No GFP-expressing neurons are seen in the bristles (arrows). The GFP signals are detected only in some non-neuronal cells (asterisks). Scale bars represent 10 μm. (0.25 MB JPG) [file pone.0011012.s002.jpg]
